# Constitutive androstane receptor and pregnane X receptor genotype influence efavirenz plasma concentration and CYP2B6 enzyme activity

**DOI:** 10.1038/s41598-022-14032-0

**Published:** 2022-06-11

**Authors:** Zelalem Petros, Abiy Habtewold, Eyasu Makonnen, Eleni Aklillu

**Affiliations:** 1 Department of Pharmacology and Clinical Pharmacy, School of Pharmacy, College of Health Sciences, Addis Ababa University, Addis Ababa, Ethiopia; 2 Department of Pharmacy, College of Medicine and Health Sciences, Wachamo University, Hosaena, Ethiopia; 3 Department of Pharmaceutical Sciences, School of Pharmacy, Wiliam Carey University, Biloxi, MS USA; 4 Center for Innovative Drug Development and Therapeutic Trials for Africa (CDT Africa), College of Health Sciences, Addis Ababa University, Addis Ababa, Ethiopia; 5 Division of Clinical Pharmacology, Department of Laboratory Medicine, Karolinska Institutet, Karolinska University Hospital-Huddinge, Stockholm, Sweden

**Keywords:** HIV infections, Tuberculosis, Combination drug therapy, Pharmacogenetics

## Abstract

Efavirenz is metabolized by CYP2B6, an inducible enzyme whose expression is regulated by the constitutive androstane receptor and pregnane X receptor nuclear receptors. CAR and PXR are encoded by genetically polymorphic *NR1I2* and *NR1I3*, respectively. We examined the impact of *NR1I2* and *NR1I3* genotype on plasma EFV concentration and CYP2B6 enzyme activity among TB-HIV co-infected patients in Ethiopia. Treatment-naïve HIV patients with TB co-infection (n = 80) were enrolled and received first-line EFV-based antiretroviral and rifampicin-based anti-TB therapy. Plasma EFV and 8-hydroxy-EFV concentrations at the 4th and 16th week of EFV treatment were determined using LC/MS/MS. EFV/8-hydroxy-EFVmetabolic ratio was used as CYP2B6 metabolic activity index. In multivariate regression analysis, *NR1I3* rs3003596C or *NR1I2* rs2472677T variant allele carriers had significantly lower plasma EFV concentrations than non-carriers. Patients with *NR1I2* rs3814057C/C genotype or *NR1I3* rs3003596C allele carriers had significantly lower mean log EFV MR. Among CYP2B6*6 allele carriers, patients with *NR1I3* rs2502815T/T or *NR1I2* rs3814057C/C genotype had significantly lower mean log EFV MR. In conclusion, genetic variants in *NR1I2* and *NR1I3* genes influence plasma EFV exposure and CYP2B6 enzyme activity in TB-HIV co-infected patients on drug treatment.

## Introduction

Tuberculosis (TB) and Human immunodeficiency virus (HIV)/Acquired Immune Deficiency Syndrome (AIDS) are diseases that cause immense burden in sub-Saharan Africa, and co-treatment of these diseases is recommended^1^. In the treatment of TB/HIV coinfections, efavirenz (EFV)-based antiretroviral therapy (ART) is the standard regimen for use together with rifampicin (RIF)-based anti-TB therapy. However, the concomitant treatment is marred by drug interactions and overlapping toxicities^2^. The plasma EFV concentration is reported to predict treatment outcomes and associated adverse events^3–6^. Sub-therapeutic plasma EFV level (< 1 μg/mL) is associated with a higher risk of treatment failure and drug resistance^3^; whereas, high plasma EFV concentration (> 4 μg/mL) increases the likelihood of developing adverse effects^6^. Thus, identifying sources of EFV pharmacokinetic variability is essential to improve therapeutic efficacy while decreasing adverse events.

EFV is primarily metabolized to 8-hydroxy-efavirenz (8-OH-EFV) by cytochrome P450 2B6 (CYP2B6) and to a lesser extent by CYP3A4^7^. Both CYP2B6 and CYP3A4 enzymes are genetically polymorphic and inducible by EFV^3^. EFV displays substantial inter-individual variations in its metabolism mainly due to genetic polymorphisms in CYP2B6 and auto-induction^8^. Our previous study on Ethiopian HIV only and TB/HIV co-treated patients also indicated the presence of wide inter-patient variability in the extent of long-term plasma EFV exposure mainly explained by CYP2B6 genotype^9^. Association of black ethnicity and CYP2B6 *c.516TT* genotype with early discontinuation of EFV containing ART regimens is reported^10^. The defective *CYP2B6c.516G* > *T*(CYP2B6*6) variant allele that impairs EFV metabolism is common in African populations^11,12^.

Constitutive androstane receptor (CAR) encoded by nuclear receptor (NR) subfamily-1 group-I member-3 (*NR1I3*) and pregnane X receptor (PXR) encoded by *NR1I2* are members of the orphan nuclear receptors (NR) super-family that function primarily as sensors of xenobiotics, and facilitate xenobiotics detoxification^13^. Upon ligand binding/activation, both PXR and CAR bind to the NR-response element in the promoter region of the target gene to regulate gene expression^14^. PXR and CAR act as master transcriptional regulators of many important genes that encode drug-metabolizing enzymes (DMEs) such as CYP3A and CYP2B^15^. Ligands that are recognized by CAR and PXR include drugs used in various clinical treatment regimens such as EFV andRIF^16^.

Several single nucleotide polymorphisms (SNPs) have been reported in *NR1I2* and *NR1I3*that are associated with changes in PXR and CARfunctions^17,18^. Variant alleles of *NR1I2*SNPs such as rs3814055 and rs6785049 were reported to have an association with altered *NR1I2* transcriptional activity^19^. A previous study that investigated the impact of polymorphisms in *NR1I2* reported significant changes in transcriptional activity associated with non-synonymous SNPs: G36R (106G > A), V140M (4374G > A), D163G (4444A > G), and A370T (8528A > G). *NR1I2* 7635A > G (rs6785049) is associated with increased expression of CYP3A4 in the presence of RIF^18^. A recent study showed the association of polymorphisms in *NR1I2* with decreased risk of RIF-based anti-TB drugs induced liver injury suggesting that drug-metabolizing enzymes regulated by PXR may be involved in the pathogenesis of anti-TB drugs induced liver injury^20^. A significant association between *NR1I3* rs2307424C > T and early EFV treatment discontinuation is reported^10^. The rs2307424 C allele carrier status was also associated with higher plasma EFV concentration in a Latin American cohort of HIV-positive patients^21^.

Genetic variations in NR1I2 and NR1I3 genes may alter the expression of CAR and PXR or their affinity to ligands, consequently variations in DMEs expressions and enzyme activity^10,22^. Hence, genetic polymorphisms in *NR1I3* and *NR1I2* genes may explain the inter-individual variability of CAR and PXR activities that affect the disposition and interaction of various drugs like EFV via an induction mechanism. Thus, genetic polymorphisms in nuclear receptor genes may influence plasma concentrations of EFV and its therapeutic efficacy and safety. Although a wide inter-individual variability in plasma EFV concentration was reported among Ethiopian TB/HIV patients^9^, the influence of SNPs in genes encoding CAR and PXR on plasma EFV concentration remains to be investigated. The current study examined the impact of genetic variations in CAR (*NR1I3*) and PXR (*NR1I2*) on plasma EFV exposure and CYP2B6 enzyme activity among TB-HIV co-infected patients on RIF-based first-line anti-TB drugs and EFV-based ART in Ethiopia.

## Methods

### Study design, participants, and drug treatments

The study design was a prospective observational pharmacokinetic and pharmacogenetic study. Participants (N = 208) were recruited from four study sites (TB/HIV clinics in Kazanchis, Beletshachew and Arada Health Centers, and Black Lion Specialized Hospital) in Addis Ababa, Ethiopia. Details of the study design, patient enrollment process, and inclusion criteria were reported previously^9^. In brief, newly diagnosed treatment naïve TB/HIV co-infected adult patients with a baseline CD4 count of ≤ 200 cells per cubic millimeter were enrolled and followed for 48 weeks. Pre-treatment laboratory tests included complete, and differential blood cell counts, HIV viral load, hepatitis B and C status, and liver and renal function tests. Patients with abnormal liver and renal function test values were excluded from the study.

Treatment was initiated following WHO and Ethiopian national guidelines for treating TB-HIV co-infections. All patients received efavirenz (EFV 600 mg once daily) and lamivudine (3TC, 150 mg twice daily) along with stavudine (d4T, 30 mg twice daily), or zidovudine (ZDV, 300 mg twice daily) or tenofovir (TDF, 300 mg once daily). Fixed-dose anti-TB treatment consisting of rifampicin (150 mg), isoniazid (75 mg), pyrazinamide (400 mg), and ethambutol (275 mg) was initiated four weeks before starting EFV-based ART. The anti-TB treatment dosage was based on the weight of the patients: 20–29 kg (1½ tablet), 30–37 (2 tablets), 38–54 kg (3 tablets) and ≥ 55 kg (4 tablets). In the study period, the participants received no other known liver microsomal enzyme inducer or inhibitor drugs concurrently.

All the study subjects provided written informed consent for participation. The study protocol got approval from the Institutional Review Board (IRB) of the School of Medicine, Addis Ababa University, and the Ethiopian National Research Ethics Review Committee. This study was conducted in accordance with the principles outlined in the Declaration of Helsinki.

### Determination of plasma EFV and 8-OH EFV concentration

On the 4th and 16th week of EFV-based ART initiation, blood samples were collected 16 h after EFV dosing in Vacutainer tubes (Becton Dickinson Heidelberg, Germany).Plasma was prepared by centrifugation (1700 g for 20 min), and aliquots were stored at − 80 °C. Determination of EFV and 8-OH-EFV concentrations was done using liquid chromatography-tandem mass spectrometry (LC/MS/MS) as described previously^8,9,23,24^. In brief, plasma proteins were precipitated with ice-cold acetonitrile. The extract underwent chromatography on a Phenomenex Synergi Fusion RP column with an eluent consisting of acidified 5 mM ammonium acetate buffer, acetonitrile, and methanol. EFV and 8-OH-EFV were quantified using ^13^C_6_-EFV and ^2^H_4_-8-OH-EFV as internal standards. The lower limit of quantification in plasma was 10 ng/mL for EFV and 0.4 ng/mL for 8-OH-EFV. The EFV and 8-OH-EFV calibration ranges were 10–10,000 ng/mL and 0.4–400 ng/mL, respectively. Linear regression with 1/X weighting resulted in correlation coefficients of r^2^ > 0.99. The assay was validated according to the FDA validation guidelines and fulfilled all the accuracy, precision, recovery, linearity, and stability criteria. EFV metabolic ratio (MR), computed by dividing the level of EFV by that of 8-OH-EFV, was used as an index of CYP2B6 activity as previously described^8^.

### SNP genotyping

Genomic DNA was isolated from peripheral blood leukocytes using QIAamp DNA Maxi Kit (QIAGEN GmbH, Hilden, Germany). Two common variant alleles from each gene: *NR1I3* (rs2502815 and rs3003596), *NR1I2* (rs3814057 and rs2472677); and a common functional CYP2B6*6(*c.516G* > *T,* rs3745274) variant alleles were investigated in the study. Genotyping was done by real-time polymerase chain reaction (PCR) using Taqman assay reagents for allelic discrimination (Applied Biosystems Genotyping Assays). Allelic discrimination reactions were performed using TaqMan genotyping assays with the following ID number for each SNP: C__16248625_10 for *NR1I3* rs2502815C > T, C__16194070_10 for *NR1I3*rs3003596T > C, C__11231739_10 for *NR1I2* rs3814057A > C, C__26079845_10 for *NR1I2*rs2472677C > T, and C__7817765_60 for *CYP2B6 c.516G* > *T* using ABI 7500 FAST Real-Time PCR Systems (Applied Biosystems, Foster City, California). The final volume for each reaction was 10 μL, consisting of TaqMan Universal PCR Master Mix (Applied Biosystems, Foster City, California), 20 × drug-metabolizing genotype assay mix, and 10 ng of genomic DNA. The PCR steps were conditioned to an initial step at 60 °C for 30 s, hold at 95 °C for 20 s, and amplifications for 40 cycles consisting of 95 °C for 1 s, 60 °C for 20 s, and a read stage at 60 °C for 30 s.

### Statistical analysis

Descriptive statistics for continuous baseline variables are presented as mean and standard deviations (SD), and categorical variables as numbers and percentages. The plasma EFV level and MR data were log-transformed to ensure the normality of the data. Chi-square test was used to compare the observed and expected allele frequencies according to the Hardy–Weinberg equilibrium and to compare the minor allele frequencies (MAFs) between the Ethiopian cohort and the corresponding allele frequencies in the HapMap reference populations of Caucasians, Asian and Yoruba origin obtained from the NCBI SNP database (dbSNP) (https://www.ncbi.nlm.nih.gov) and literature^25^. Univariate regression analysis was used to identify potential independent variables affecting the continuous outcome variables. Independent variables with *P* ≤ 0.10 in the univariate analysis were included in the multivariate regression analysis. Bonferroni's correction was applied for multiple comparisons to account for the number of SNPs investigated. Statistical analysis was performed using SPSS Statistics for Windows, Version 20.0 (IBM Corp., Armonk, NY, USA), and *P* < 0.05 was considered as significant.

## Results

### Clinical characteristics of the study participants

In this study, 80 TB-HIV co-infected patients were involved, and plasma EFV pharmacokinetic data were determined at the 4th and 16th weeks of initiating EFV-based ART. The Sociodemographic, baseline clinical, and biochemical parameters of the study participants are presented in Table 1.

**Table 1:** Baseline demographic, clinical and laboratory characteristics of study participants [3TC—Lamivudine; D4T—Stavudine; EFV—Efavirenz; N—Number; SD—Standard deviation; TDF—Tenofovir; ZDV—Zidovudine].

Parameters	Values
Gender, N (%)	
Male	46 (57.5)
Female	34 (42.5)
Karnofsky score, N (%)	
90 and 100%	42 (52.5)
70 and 80%	32 (40.0)
50 and 60%	6 (7.5)
Type of antiretroviral regmine, N (%)	
EFV + 3TC + D4T	38.0 (47.5)
EFV + 3TC + ZDV	28.0 (35.0)
EFV + 3TC + TDF	14.0 (17.5)
Age in years, Mean (SD)	35.7 (8.8)
Body mass index (kg/m^2^), Mean (SD)	18.4 (2.4)
Hepatitis B virus surface antigen N (%)	5.0 (6.3)
Anti-hepatitis C virus antibody N (%)	1.0 (1.3)
Hemoglobin (gm/dL), Mean (SD)	11.4 (2.4)
WBC count (× 10^3^ per mL), Mean (SD)	5.9 (2.5)
Platelets (× 10^3^ per mL), Mean (SD)	290.0 (129.0)
Neutrophils (%),Mean (SD)	65.0 (14.0)
CD4 count (per mm^3^), Mean (SD)	91.0 (52.0)
HIV viral load (copies/mL), log Mean (SD)	5.1 (0.8)
Alanine aminotransferase (U/L), Mean (SD)	40.0 (37.0)
Aspartate aminotransferase (U/L), Mean (SD)	58.0 (39.0)
Alkaline phosphatase (U/L), Mean (SD)	109.0 (59.0)
Total bilirubin (mg/dL), Mean (SD)	0.7 (0.6)
Plasma creatinine (μmol/L), Mean (SD)	0.9 (0.2)
Albumin (gm/dL), Mean (SD)	3.8 (2.4)
Urea (mg/dL), Mean (SD)	27.0 (10.0)

### Minor allele frequency (MAF)

The observed genotype frequency distributions of all the SNPs investigated in the current study were consistent with Hardy–Weinberg equilibrium. The MAFs of *NR1I3*, *NR1I2*, and *CYP2B6* variant alleles in the study cohort (Ethiopian TB-HIV co-infected patients) and the HapMap reference populations of Caucasians, Asian and Yoruba origin obtained from the NCBI SNP database (dbSNP) are shown in Table 2. There were statistically significant differences in the MAFs of (a) *NR1I3* rs2502815 and *NR1I2* rs3814057 between the Ethiopian cohort and the corresponding MAFs in the European cohort, (b) *NR1I2* rs2472677 and CYP2B6*6 (rs3745274) between the Ethiopian cohort and the corresponding MAF in the Yoruba cohort.

**Table 2:** Comparison of minor allele frequencies between the study cohort and the HapMap reference populations [^*^ Ethiopians; CEU—Utah residents of Northern and Western European ancestry; HCB—Han Chinese from Beijing, China; JPT—Japanese from Tokyo, Japan; MAF—Minor allele frequency in the study cohort; N—Sample size; YRI—Yoruba trios from Ibadan, Nigeria].

Population	NR1I3 rs2502815C > T			NR1I3 rs3003596T > C			NR1I2 rs3814057A > C			NR1I2 rs2472677C > T			CYP2B6 rs3745274G > T		
	N	C (MAF)	*P*	N	T (MAF)	*P*	N	C (MAF)	*P*	N	C (MAF)	*P*	N	T (MAF)	*P*
Study cohort*	80	0.49	–	80	0.48	–	80	0.41	–	80	0.43	–	80	0.27	–
CEU	226	0.75	**< 0.01**	118	0.59	0.11	226	0.09	**< 0.01**	118	0.36	0.37	226	0.27	1.00
HCB	86	0.56	0.44	86	0.47	1.00	80	0.45	0.75	90	0.46	0.76	86	0.15	0.06
JPT	172	0.54	0.50	88	0.36	0.16	88	0.52	0.17	90	0.42	1.00	172	0.18	0.92
YRI	224	0.59	0.12	116	0.39	0.24	222	0.49	0.24	118	0.64	** < 0.01**	224	0.42	**0.02**

Significant values are in [bold].

### Effect of NR1I3 and NR1I2genotypeson plasma EFV level and metabolic ratios

In multivariate regression analysis at week-4 (Table 3), *NR1I3* rs3003596*T/T* genotype was associated with significantly higher mean log plasma EFV concentration compared to the *NR1I3*rs3003596*C/C* and *C/T* genotypes together (*P* = 0.03) at week-4. Two of the twenty-two (9.1%) patients with rs3003596C/C genotype had mean log plasma EFV concentrations above 4 μg/mL, whereas six (35.3%) patients with the rs3003596T/T genotype had mean log plasma EFV concentrations above 4 μg/mL. The *NR1I3* rs3003596C allele carriers had reduced mean log plasma EFV concentrations compared to the allele non-carriers. *NR1I2* rs2472677C/C genotype was also associated with significantly higher mean log plasma EFV concentration compared to *NR1I2* rs2472677C/T and T/T genotypes together at week-4 (*P* = 0.04) and at week-16 (*P* = 0.02). The *NR1I2* rs2472677T allele carriers had lower plasma EFV concentrations compared to the allele non-carriers.

**Table 3:** Regression analysis for *NR1I3* and *NR1I2* variants on mean log plasma EFV concentrations at week-4 and week-16 after initiation of EFV-based ART [^*^—Mean of log plasma EFV concentrations (ng/mL) and the standard deviation (SD); ART—Antiretroviral therapy; CI—Confidence interval; EFV—Efavirenz; N—Number; *P*—*P*-value].

Variables		Week-4						Week-16					
		N	Mean ± SD*	Univariate Exp β (95% CI)	*P*	Multivariate Exp β (95% CI)	*P*	N	Mean ± SD*	Univariate Exp β (95% CI)	*P*	Multivariate Exp β (95% CI)	*P*
Sex	Male	41	3.16 ± 0.34	1.08 (0.91–1.28)	0.38			33	3.13 ± 0.31	1.09 (0.91–1.31)	0.30		
	Female	29	3.23 ± 0.38					24	3.23 ± 0.36				
*NR1I3* rs2502815	T/T	16	3.12 ± 0.41	1.08 (0.89–1.34)	0.41			16	3.06 ± 0.33	1.17 (0.96–1.42)	0.12		
	C/T + C/C	54	3.21 ± 0.34					41	3.22 ± 0.33				
*NR1I3* rs3003596	T/T	17	3.35 ± 0.36	0.80 (0.66–0.98)	**0.03**	0.80 (0.66–0.97)	**0.03**	12	3.23 ± 0.35	0.93 (0.75–1.16)	0.51		
	C/T + C/C	53	3.14 ± 0.34					45	3.16 ± 0.33				
*NR1I2* rs3814057	C/C	10	3.10 ± 0.20	0.90 (0.70–1.15)	0.39			8	2.97 ± 0.25	0.79 (0.62–1.02)	0.07	0.82 (0.66–1.04)	0.10
	A/C + A/A	60	3.20 ± 0.38					49	3.21 ± 0.36				
*NR1I2* rs2472677	C/C	13	3.36 ± 0.34	0.81 (0.65–1.01)	0.05	0.81 (0.66–0.99)	**0.04**	7	3.39 ± 0.23	0.78 (0.60–1.01)	0.06	0.73 (0.57–0.96)	**0.02**
	C/T + T/T	57	3.15 ± 0.35					50	3.14 ± 0.34				

Significant values are in [bold].

In multivariate analysis at week-4 (Table 4), *NR1I3* rs3003596T/T genotype was associated with significantly higher mean log EFV MR compared to the *NR1I3* rs3003596C/T and C/C genotypes together (*P* = 0.01). The *NR1I2* rs3814057C/C genotype was also associated with significantly reduced mean log EFV MR compared to *NR1I2* rs3814057A/C and A/A genotypes together (*P* = 0.002). This association remained significant after Bonferroni's correction for multiple testing (*P* < 0.01). There was no statistically significant difference in the mean log EFV MR in *NR1I3* rs2502815T/T genotype compared to *NR1I3* rs2502815C/C and C/T genotypes together at week 4. However, in multivariate analysis at week-16, *NR1I3* rs2502815T/T genotype was associated with reduced mean log EFV MR compared to *NR1I3* rs2502815C/C and T/C genotypes together (*P* = 0.048).

**Table 4:** Regression analysis for *NR1I3* and *NR1I2*variants on mean log plasma EFV/8-OH-EFV metabolic ratios (MRs) at week-4 and week-16 after initiation of EFV-based ART [^*^—Mean of log plasma EFV Metabolic ratios and the standard deviation (SD); ART—Antiretroviral therapy; CI—Confidence interval; EFV—Efavirenz; MR—Metabolic ratio; N—Number; *P*—*P* value].

Variables		Week-4						Week-16					
		N	EFV MR Mean ± SD*	Univariate Exp β (95% CI)	*P*	Multivariate Exp β (95% CI)	*P*	N	EFV MR Mean ± SD*	Univariate Exp β (95% CI)	*P*	Multivariate Exp β (95% CI)	*P*
Sex	Male	41	1.31 ± 0.52	0.94 (0.73–1.21)	0.64			33	1.39 ± 0.48	0.87 (0.68–1.11)	0.24		
	Female	29	1.25 ± 0.53					24	1.24 ± 0.41				
*NR1I3* rs2502815	T/T	16	1.13 ± 0.45	1.22 (0.90–1.63)	0.18			16	1.13 ± 0.29	1.32 (1.02–1.72)	**0.04**	1.30 (1.01–1.67)	**0.048**
	C/T + C/C	54	1.33 ± 0.54					41	1.40 ± 0.48				
*NR1I3* rs3003596	T/T	17	1.50 ± 0.60	0.75 (0.56–0.99)	**0.04**	0.70 (0.53–0.91)	**0.01**	12	1.39 ± 0.57	0.92 (0.69–1.25)	0.61		
	C/T + C/C	53	1.21 ± 0.48					45	1.31 ± 0.42				
*NR1I2* rs3814057	C/C	10	0.87 ± 0.30	0.62 (0.44–0.87)	**0.007**	0.58 (0.44–0.83)	**0.002**	8	1.04 ± 0.29	0.72 (0.51–1.01)	0.06	0.74 (0.53–1.03)	0.07
	A/C + A/A	60	1.35 ± 0.52					49	1.37 ± 0.46				
*NR1I2* rs2472677	C/C	13	1.49 ± 0.54	0.77 (0.56–1.06)	0.11			7	1.25 ± 0.31	1.08 (0.75–1.57)	0.66		
	C/T + T/T	57	1.23 ± 0.51					50	1.34 ± 0.47				

Significant values are in [bold].

### Effect of NR1I3 and NR1I2genotype on EFV MR stratified by CYP2B6*6 carrier status

Among CYP2B6*6 allele carriers, in multivariate analysis (Tables 5 and 6), the *NR1I2* rs3814057C/C genotype was associated with significantly reduced mean log EFV MR compared to *NR1I2* rs3814057A/C and A/A genotypes together (*P* = 0.011) at week 4. This association remained significant after Bonferroni's correction for multiple testing. The*NR1I3* rs2502815T/T genotype was also associated with significantly reduced mean log EFV MR compared to the *NR1I3* rs2502815C/C and T/C genotypes (*P* = 0.046). At week 16, *NR1I3*rs2502815T/T, genotype was associated with significantly reduced mean log EFV MR compared to *NR1I3*rs2502815 C/T and C/C genotypes together (*P* = 0.02). Among CYP2B6*6 allele non-carriers, none of the variants investigated was associated with the mean log EFV MR at both study time points.

**Table 5:** Regression analysis for *NR1I3*, *NR1I2* variants on mean log EFV/8-OH-EFV metabolic ratios (MRs) at week-4 after initiation of EFV-based ART, stratified with CYP2B6*6 carrier status [^*^—Mean of log plasma EFV Metabolic ratios and with the standard deviation (SD); ART—Antiretroviral therapy; CI—Confidence interval; EFV—Efavirenz; MR—Metabolic ratio; N—Number; *P*—*P* value].

Genotypes		CYP2B6*6 allele carriers						CYP2B6*6 allele non-carriers			
		N	Mean ± SD*	Univariate Exp β (95% CI)	*P*	Multivariate Exp β (95% CI)	*P*	N	Mean ± SD*	Univariate Exp β (95% CI)	*P*
NR1I3 rs2502815	T/T	5	1.01 ± 0.43	1.82 (1.03–3.19)	**0.04**	1.70 (1.01–2.86)	**0.046**	11	1.19 ± 0.46	0.88 (0.68–1.13)	0.31
	C/T + C/C	27	1.60 ± 0.59					27	1.05 ± 0.30		
NR1I3 rs3003596	T/T	12	1.65 ± 0.61	0.80 (0.51–1.26)	0.32			5	1.16 ± 0.46	0.92 (0.66–1.31)	0.67
	C/T + C/C	20	1.42 ± 0.60					33	1.08 ± 0.34		
NR1I2 rs3814057	C/C	4	0.80 ± 0.29	0.44 (0.24–0.80)	**0.009**	0.47 (0.27–0.83)	**0.01**	6	0.92 ± 0.33	0.82 (0.59–1.13)	0.21
	A/C + A/A	28	1.61 ± 0.57					32	1.12 ± 0.35		
NR1I2 rs2472677	C/C	6	1.90 ± 0.49	0.62 (0.36–1.05)	0.08	0.76 (0.46–1.23)	0.25	7	1.15 ± 0.29	0.93 (0.69–1.27)	0.66
	C/T + T/T	26	1.42 ± 0.60					31	1.08 ± 0.37		

Significant values are in [bold].

**Table 6:** Regression analysis for *NR1I3*, *NR1I2* variants on mean log EFV/8-OH-EFV metabolic ratios (MRs) at week-16 after initiation of EFV-based ART, stratified with CYP2B6*6 carrier status [^*^—Mean of log plasma EFV Metabolic ratios and with the standard deviation (SD); ART—Antiretroviral therapy; CI—Confidence interval; EFV—Efavirenz; MR—Metabolic ratio; N—Number; *P*—*P* value].

Genotypes		CYP2B6*6 allele carriers						CYP2B6*6 allele non-carriers			
		N	Mean ± SD*	Univariate Exp β (95% CI)	*P*	Multivariate Exp β (95% CI)	*P*	N	Mean ± SD*	Univariate Exp β (95% CI)	*P*
NR1I3 rs2502815	T/T	5	1.20 ± 0.15	0.65 (0.47–0.97)	**0.004**	2.30 (1.94–4.18)	**0.02**	11	1.09 ± 0.34	0.37 (0.12–1.37)	0.98
	C/T + C/C	26	1.58 ± 0.49					15	1.09 ± 0.27		
NR1I3 rs3003596	T/T	9	1.55 ± 0.57	0.42 (0.21–1.12)	0.87			23	1.11 ± 0.30	0.42 (0.27–1.75)	0.26
	C/T + C/C	22	1.51 ± 0.44					3	0.91 ± 0.21		
NR1I2 rs3814057	C/C	3	1.24 ± 0.39	0.81 (0.22–1.41)	0.29			5	0.92 ± 0.16	0.20 (0.12–1.51)	0.16
	A/C + A/A	28	1.55 ± 0.48					21	1.13 ± 0.31		
NR1I2 rs2472677	C/C	2	1.53 ± 0.49	0.54 (0.42–1.08)	0.82			5	1.18 ± 0.31	1.21 (0.70–1.78)	0.48
	C/T + T/T	29	1.45 ± 0.31					21	1.07 ± 0.30		

Significant values are in [bold].

## Discussion

This study investigated the impact of genetic variations in CAR (*NR1I3*) and PXR (*NR1I2*) genes on variability in plasma EFV exposure and CYP2B6 metabolic activity among TB-HIV co-infected patients on concomitant first-line anti-TB and EFV-based ART in Ethiopia. EFV/8-OH-EFV metabolic ratio was used as a marker for CYP2B6 enzyme activity. The main findings indicate that patients with *NR1I3* rs3003596C and *NR1I2* rs2472677T carriers had significantly reduced plasma EFV concentrations. In addition, patients with *NR1I2* rs3814057C/C genotype or *NR1I3* rs3003596C allele carriers had significantly lower mean log EFV MRs. Among CYP2B6*6 allele carrier patients, *NR1I2* rs3814057C/C or *NR1I3* rs2502815T/T genotype displayed significantly lower mean log EFV MRs. Among CYP2B6*6 allele non-carriers, none of the variants were associated with the mean log EFV MR. To our knowledge, this is the first report to investigate the impact of *NR1I2* and *NR1I3* genotypes on CYP2B6 activity using EFV metabolic ratio as a marker among the TB-HIV co-infected patients.

PXR and CAR act as master transcriptional regulators of many genes that encode DMEs such as CYP3A and CYP2B enzymes^15^. Ligands that are recognized by CAR and PXR include drugs used in various clinical treatment regimens such as EFV, RIF and others^16^. The current study showed significant associations of genetic variations in PXR and CAR with variability in EFV pharmacokinetics and CYP2B6 metabolic activity. This may have clinical implications in influencing the therapeutic efficacy and safety of EFV containing regimens. Our study finding highlights the potential relevance of CAR and PXR genotype in altering metabolic regulation of DMEs, and hence the pharmacokinetics and treatment outcomes of drugs through nuclear-receptor mediated pathways.

The *NR1I3* and *NR1I2* gene variants investigated in this study displayed significant differences in MAF between the Ethiopian cohort and at least one of the HapMap reference populations. As the SNPs are associated with plasma EFV concentration and MR, the allele frequency difference among the populations may result in between-populations variation in EFV pharmacokinetics. Likewise, African populations cannot be regarded as homogeneous due to the genetic diversity existing between the sub-populations. For example, *NR1I2*rs2472677C > T and *CYP2B6* rs3745274G > T showed a significant difference in MAF between the Ethiopian cohort and the Yoruba population from Ibadan, Nigeria. This further substantiates the need for population-specific pharmacogenetic studies in Africans to identify genetic markers for altered pharmacokinetics of drugs. The black African population is the most genetically heterogeneous population globally, characterized by extensive population substructure and low linkage disequilibrium (LD) among genomic loci compared to non-African populations^26,27^. HIV and TB infections remain major problems in sub-Saharan Africa, and treatment has been scaled up. Hence, the identification of genetic biomarkers that predict the pharmacokinetics of ART and anti-TB drugs in different black African populations is important.

In this study, the SNPs investigated for their association with mean log plasma EFV concentrations and MRs were selected based on previous reports of high MAF in African-American and other black African populations^25^, and evidence of being functionally associated with altered expression levels, modified regulation of downstream effector genes involved in xenobiotic removal or potential to affect altered expression by virtue of its location in regulatory regions or transcription factor binding sites^17,19,28^. Of the four SNPs examined in *NR1I3* and *NR1I2* genes, *NR1I3* rs2502815C > T, *NR1I3* rs3003596T > C, and *NR1I2* rs2472677C > T are intronic polymorphisms, whereas *NR1I2* rs3814057A > C is located in the 3'-untranslated region (3'UTR). Emerging evidence indicates that non-coding genetic variants play an important role in gene regulation by influencing the transcriptional activity, splicing efficiency, or by altering the splicing site of their host genes^29^. Thus, the SNPs found to have an association with the mean log plasma EFV levels and MR in the current study might affect the transcriptional activity or efficiency of *NR1I3* and *NR1I2* genes.

Interestingly, we found significantly lower mean log plasma EFV concentrations and MRs among patients with *NR1I3* rs3003596C/C and C/T genotypes compared to patients with the *NR1I3* rs3003596T/T genotype, similar to what has been reported by Swart et al.^25^. This finding is also in line with other previous studies that implicated *NR1I3* rs3003596 in altered EFV concentrations or treatment outcomes^10,21^. In our study, there was also a lower mean log EFV MR observed among patients with *NR1I3* rs2502815T/T genotype compared to patients with the *NR1I3* rs2502815C/C and C/T genotypes. Swart et al*.* also reported that the *NR1I3* rs2502815T/T genotype was associated with reduced plasma EFV concentration although not statistically significant^25^. In the current study, the low mean log plasma EFV concentrations and MRs in *NR1I3* rs3003596C and *NR1I3* rs2502815T variant carriers may indicate possible functional effects of the change on CAR expression or activity and regulation of target genes encoding DME such as CYP2B6. This observation aligns with the hypothesis that variation in CAR activity alters CYP2B6 activity and, therefore, change in EFV exposure. EFV induces CYP2B6 enzyme activity primarily through CAR^30^, and the associated genetic variants in *NR1I3* may thus significantly influence plasma EFV concentrations via the induction of CYP2B6. Therefore, *NR1I3* rs3003596C and *NR1I3* rs2502815T variants may be associated with a gain of function (increased expression of CYP2B6) rather than a loss.

Associations between variants in CAR and EFV pharmacokinetics have been linked to the regulation of CYP2B6 expression by CAR^30^. Chang et al*.* reported more than 200-fold inter-individual variability and a positive correlation between hepatic CAR and CYP2B6 mRNA expression^31^. Research findings suggest that upon activation, CAR binds to a proximal-responsive enhancer module (PREM) located approximately 2 kb upstream from the CYP2B gene transcriptional start site as a heterodimer with retinoid X receptor to influence gene expression^32^. CAR, which also binds to a xenobiotic-responsive enhancer module (XREM) in the distal region of the CYP2B6 promoter together with the PREM, mediates optimal drug-induced expression of CYP2B6^32,33^.

In the present study, significantly reduced mean log plasma EFV levels were observed among patients with *NR1I2* rs2472677T/T and C/T genotypes compared to patients with the *NR1I2* rs2472677C/C genotype. There was also significantly low mean log EFV MR observed among patients with *NR1I2* rs3814057C/C genotype compared to patients with the *NR1I2* rs3814057A/C and A/A genotypes. By activating CAR alone or in cross-talk to PXR, EFV enhances the expression of multiple enzymes regulated by these NRs, including CYP2B6, CYP3A4, and CYP2A6^31,34,35^. The *NR1I2* rs2472677T variant and rs3814057C/C genotype associated with reduced log plasma EFV level and MR could possibly act through increased PXR and/or CAR transcription and thus the increased transcription of downstream DME genes such as CYP3A4 and CYP2B6 leading to increased EFV clearance.

Siccardi et al*.* reported that *NR1I2* rs2472677 variants are associated with altered antiretroviral plasma concentrations^36^. Patients homozygous for *NR1I2* rs2472677T variant had increased CYP3A4 expression resulting in decreased plasma atazanavir concentration below the minimum effective level. The proposed mechanism of altering downstream DME genes in this study and our finding on the association of *NR1I2* rs3814057 variants with mean log plasma EFV MR are also consistent with a previous study where analysis of CYP3A4 enzyme activity in the presence of RIF identified rs3814055, which is in LD with *NR1I2*rs3814057 and significantly associated with increased CYP3A4 activity^17^.

We further explored the importance of treatment duration for the effect of PXR and CAR genotype on plasma EFV concentration and its metabolic ratio. The effect of PXR genotype on plasma EFV concentration was significant beyond week 4, as indicated by the significant association of NR1I2 rs2472677C > T genotype with plasma EFV concentration at week-16 (Table 3). But the influence of CAR genotype on plasma EFV concentration was significant only at week 4. On the other hand, while the influence of PXR genotype on plasma EFV MR was significant only at week 4, the influence of CAR genotype on EFV MR continued beyond week 4, as indicated by the statistically significant effect of NR1I3 rs2502815T > C genotype on EFV MR at week-16 (Table 4). This shows the importance of treatment duration on efavirenz-rifampicin drug interactions, and the relevance of PXR and CAR in influencing plasma EFV concentration and EFV MR, respectively.

Previously, we reported that the mean log plasma EFV concentrations and MRs in CYP2B6*6 allele carriers is significantly higher than the allele non-carrirers^8^. In the current study, *NR1I3* rs2502815T/T and *NR1I2* rs3814057C/C genotypes were associated with significantly decreased mean log EFV MR among carriers of the defective CYP2B6*6 allele. This might indicate the possible increase in *NR1I3* and *NR1I2* expressions and activities in the presence of the variants, eliciting effects through other enzymes that participate in EFV metabolism, most likely CYP3A enzymes. In CYP2B6*6 allele non-carriers with intact CYP2B6 enzyme activity, *NR1I3* and *NR1I2* gene variants were not associated with changes in the mean log EFV MR. EFV is primarily metabolized to 8-OH-EFV by CYP2B6, and to a lesser extent by CYP3A4 and CYP2A6^7^. The influence of the other enzymes such as CYP3A4 on EFV metabolism might not be strong enough in the presence of intact CYP2B6 enzyme activity, resulting in a non-significant association of the SNPs in CYP2B6*6 allele non-carriers. The importance of CYP3A4 in mediating EFV metabolism, particularly in subjects with CYP2B6 slow metabolizers, is described previously^11^. Thus, the significant reduction of mean log EFV MR in CYP2B6*6 allele carriers could partly be explained by increased CYP3A4 enzyme activity through increased PXR and CAR expression in polymorphic *NR1I3* and *NR1I2* genes. This finding for the impact of *NR1I3* and *NR1I2* gene polymorphisms on metabolic activity in CYP2B6*6 allele carriers requires further assessment.

In the univariate analysis in the CYP2B6*6 allele carriers, we observed that the association of *NR1I2* (PXR) rs3814057 with mean log EFV MR remained significant whereas the effect of *NR1I3* (CAR) rs2502815 was not significant after correction for multiple testing. This might be in agreement with the report that PXR increases the activity of CYP3A4 to a large extent while CAR increases its activity to a limited extent^37^.

Gatanaga et al*.* investigated the potential clinical application of pharmacogenetics in optimizing EFV-based ART, and EFV was initiated at a reduced daily dose in patients with CYP2B6 516G > T genotype status^38^. In that study, adequate plasma exposure and sustained virological suppression were achieved, and central nervous system-related symptoms were improved in more than two-third of the patients. However, the high plasma EFV concentration despite dose reduction in some individuals with CYP2B6*6/*6 genotype in that study suggests the need to consider other possible factors for optimization of the treatment. As shown from our study findings, considering specific polymorphisms in *NR1I3* and *NR1I2* genes together with variation in EFV metabolizing genes, primarily CYP2B6, may have significant clinical relevance in designing the genotyping assays for individualized EFV dose, and for monitoring efficacy and patient safety.

Rifampicin (RIF) is a known liver microsomal enzyme inducer, which induces CYP enzyme by activating NRs^39,40^. As RIF induces CYP2B6 and CYP3A4, concomitant RIF-based anti-TB treatment is expected to increase the apparent clearance of EFV, which is the substrate of these iso-enzymes^40^. However, a previous study by our group showed no significant difference in long-term plasma EFV pharmacokinetic parameters between TB/HIV co-treated patients and those treated for HIV only^23^. In the current study on TB/HIV co-infected patients on concomitant first-line anti-TB and EFV-bases ART in Ethiopia, the association of specific *NR1I3* and *NR1I2* gene polymorphisms on EFV pharmacokinetics were observed. We recommend evaluating the associated SNPs on plasma EFV concentration and MR in HIV patients on EFV-based ART without anti-TB treatment.

There were some limitations in the current study. First, the sample size for the sub-group analysis was small, particularly patients with homozygous CYP2B6*6/*6 genotype (n = 32). However, we confidently inferred that the effects of *NR1I3* and *NR1I2* genotypes on the mean log EFV MR are not significant for CYP2B6*6 allele non-carriers (n = 38). The impact of *NR1I3* and *NR1I2* gene variants on metabolic activity in CYP2B6*6 allele carriers needs to be replicated in a larger sample size study. Second, we did not examine all the known functional polymorphisms of CAR and PXR; instead, we focused on common SNPs that are frequently found in black African populations. However, we detected a significant impact of the selected SNPs on EFV pharmacokinetics and CYP2B6 metabolic activity.

## Conclusions

Our findings reveal a significant association of specific *NR1I3* and *NR1I2* genotypes with variability in EFV pharmacokinetics and CYP2B6 metabolic activity, as measured by the mean plasma EFV concentration and metabolic ratio. This study provides clinically relevant information on the impact of *NR1I3* and *NR1I2* genotype in affecting CYP2B6 enzyme activity and plasma efavirenz exposure, hence its therapeutic efficacy and safety.

## Data Availability

All data generated or analyzed in this study are included in this published article, and the datasets are available from the corresponding author within limits imposed by ethical and legal dispositions.

## References

[1] Harries AD, Zachariah R, Lawn SD (2009). Providing HIV care for co-infected tuberculosis patients: A perspective from sub-Saharan Africa. Int. J. Tuberc. Lung Dis. Off. J. Int. Union Against Tuberc. Lung Dis..

[2] Egelund EF, Dupree L, Huesgen E, Peloquin CA (2017). The pharmacological challenges of treating tuberculosis and HIV coinfections. Expert Rev. Clin. Pharmacol..

[3] Mukonzo JK (2013). Influence of efavirenz pharmacokinetics and pharmacogenetics on neuropsychological disorders in Ugandan HIV-positive patients with or without tuberculosis: A prospective cohort study. BMC Infect. Dis..

[4] Yimer G (2012). High plasma efavirenz level and CYP2B6*6 are associated with efavirenz-based HAART-induced liver injury in the treatment of naive HIV patients from Ethiopia: A prospective cohort study. Pharmacogenomics J..

[5] Yimer G (2011). Pharmacogenetic & pharmacokinetic biomarker for efavirenz based ARV and rifampicin based anti-TB drug induced liver injury in TB-HIV infected patients. PLoS ONE.

[6] Marzolini C (2001). Efavirenz plasma levels can predict treatment failure and central nervous system side effects in HIV-1-infected patients. AIDS.

[7] Ward BA (2003). The cytochrome P450 2B6 (CYP2B6) is the main catalyst of efavirenz primary and secondary metabolism: Implication for HIV/AIDS therapy and utility of efavirenz as a substrate marker of CYP2B6 catalytic activity. J. Pharmacol. Exp. Ther..

[8] Habtewold A (2011). Long-term effect of efavirenz autoinduction on plasma/peripheral blood mononuclear cell drug exposure and CD4 count is influenced by UGT2B7 and CYP2B6 genotypes among HIV patients. J. Antimicrob. Chemother..

[9] Habtewold A (2015). Is there a need to increase the dose of efavirenz during concomitant rifampicin-based antituberculosis therapy in sub-Saharan Africa? The HIV-TB pharmagene study. Pharmacogenomics.

[10] Wyen C (2011). Cytochrome P450 2B6 (CYP2B6) and constitutive androstane receptor (CAR) polymorphisms are associated with early discontinuation of efavirenz-containing regimens. J. Antimicrob. Chemother..

[11] Ngaimisi E (2010). Long-term efavirenz autoinduction and its effect on plasma exposure in HIV patients. Clin. Pharmacol. Ther..

[12] Mukonzo JK (2009). A novel polymorphism in ABCB1 gene, CYP2B6*6 and sex predict single-dose efavirenz population pharmacokinetics in Ugandans. Br. J. Clin. Pharmacol..

[13] Mbatchi LC, Brouillet J-P, Evrard A (2018). Genetic variations of the xenoreceptors NR1I2 and NR1I3 and their effect on drug disposition and response variability. Pharmacogenomics.

[14] Buchman CD, Chai SC, Chen T (2018). A current structural perspective on PXR and CAR in drug metabolism. Expert Opin. Drug Metab. Toxicol..

[15] Whyte-Allman SK, Hoque MT, Jenabian MA, Routy JP, Bendayan R (2017). Xenobiotic nuclear receptors pregnane X receptor and constitutive androstane receptor regulate antiretroviral drug efflux transporters at the blood-testis barrier. J. Pharmacol. Exp. Ther..

[16] di Masi A, De Marinis E, Ascenzi P, Marino M (2009). Nuclear receptors CAR and PXR: Molecular, functional, and biomedical aspects. Mol. Asp. Med..

[17] Lamba J, Lamba V, Strom S, Venkataramanan R, Schuetz E (2008). Novel single nucleotide polymorphisms in the promoter and intron 1 of human pregnane X receptor/NR1I2 and their association with CYP3A4 expression. Drug Metab. Dispos. Biol. Fate Chem..

[18] Hustert E (2001). Natural protein variants of pregnane X receptor with altered transactivation activity toward CYP3A4. Drug Metab. Dispos. Biol. Fate Chem..

[19] Zhang J (2001). The human pregnane X receptor: Genomic structure and identification and functional characterization of natural allelic variants. Pharmacogenetics.

[20] Wang Y (2019). Association of PXR and CAR polymorphisms and antituberculosis drug-induced hepatotoxicity. Sci. Rep..

[21] Cortes CP (2013). Correlates of efavirenz exposure in Chilean patients affected with human immunodeficiency virus reveals a novel association with a polymorphism in the constitutive androstane receptor. Ther. Drug Monit..

[22] Chung JY (2011). Effects of pregnane X receptor (NR1I2) and CYP2B6 genetic polymorphisms on the induction of bupropion hydroxylation by rifampin. Drug Metab. Dispos. Biol. Fate Chem..

[23] Habtewold A (2016). Long-term effect of rifampicin-based Anti-TB Regimen coadministration on the pharmacokinetic parameters of efavirenz and 8-Hydroxy-efavirenz in ethiopian patients. J. Clin. Pharmacol..

[24] Ngaimisi E (2013). Importance of ethnicity, CYP2B6 and ABCB1 genotype for efavirenz pharmacokinetics and treatment outcomes: A parallel-group prospective cohort study in two sub-Saharan Africa populations. PLoS ONE.

[25] Swart M (2012). PXR and CAR single nucleotide polymorphisms influence plasma efavirenz levels in South African HIV/AIDS patients. BMC Med. Genet..

[26] Campbell MC, Tishkoff SA (2008). African genetic diversity: Implications for human demographic history, modern human origins, and complex disease mapping. Annu. Rev. Genom. Hum. Genet..

[27] Teo Y, Small K, Kwiatkowski D (2010). Methodological challenges of genome-wide association analysis in Africa. Nat. Rev. Genet..

[28] Svard J, Spiers JP, Mulcahy F, Hennessy M (2010). Nuclear receptor-mediated induction of CYP450 by antiretrovirals: Functional consequences of NR1I2 (PXR) polymorphisms and differential prevalence in whites and sub-Saharan Africans. J. Acquir. Immune Defic. Syndr..

[29] Cooper DN (2010). Functional intronic polymorphisms: Buried treasure awaiting discovery within our genes. Hum Genomics.

[30] Sueyoshi T, Negishi M (2001). Phenobarbital response elements of cytochrome P450 genes and nuclear receptors. Annu. Rev. Pharmacol. Toxicol..

[31] Chang TK, Bandiera SM, Chen J (2003). Constitutive androstane receptor and pregnane X receptor gene expression in human liver: Interindividual variability and correlation with CYP2B6 mRNA levels. Drug Metab. Dispos. Biol. Fate Chem..

[32] Thorn CF, Lamba JK, Lamba V, Klein TE, Altman RB (2010). PharmGKB summary: Very important pharmacogene information for CYP2B6. Pharmacogenet. Genomics.

[33] Wang H (2003). A novel distal enhancer module regulated by pregnane X receptor/constitutive androstane receptor is essential for the maximal induction of CYP2B6 gene expression. J. Biol. Chem..

[34] Hariparsad N (2004). Induction of CYP3A4 by efavirenz in primary human hepatocytes: Comparison with rifampin and phenobarbital. J. Clin. Pharmacol..

[35] Faucette SR (2007). Relative activation of human pregnane X receptor versus constitutive androstane receptor defines distinct classes of CYP2B6 and CYP3A4 inducers. J. Pharmacol. Exp. Ther..

[36] Siccardi M (2008). Association of a single-nucleotide polymorphism in the pregnane X receptor (PXR 63396C>T) with reduced concentrations of unboosted atazanavir. Clin. Infect. Dis. Off. Publ. Infect. Dis. Soc. Am..

[37] Wang YM, Ong SS, Chai SC, Chen T (2012). Role of CAR and PXR in xenobiotic sensing and metabolism. Expert Opin. Drug Metab. Toxicol..

[38] Gatanaga H (2007). Successful efavirenz dose reduction in HIV type 1-infected individuals with cytochrome P450 2B6 *6 and *26. Clin. Infect. Dis. Off. Publ. Infect. Dis. Soc. Am..

[39] Yenny, Y. Pharmacokinetic interactions between rifampicin and efavirenz in HIV-TB coinfections. *Univ. Med.* **28**, 188–201 (2009).

[40] Gengiah TN (2012). The influence of tuberculosis treatment on efavirenz clearance in patients co-infected with HIV and tuberculosis. Eur. J. Clin. Pharmacol..

